# Regulation and roles of bicarbonate transporters in cancer

**DOI:** 10.3389/fphys.2014.00130

**Published:** 2014-04-16

**Authors:** Andrej Gorbatenko, Christina W. Olesen, Ebbe Boedtkjer, Stine F. Pedersen

**Affiliations:** ^1^Department of Biology, University of CopenhagenCopenhagen, Denmark; ^2^Department of Biomedicine, Aarhus UniversityAarhus, Denmark

**Keywords:** NBCn1, DRA, ion transport, acid-base regulation, intracellular pH

## Abstract

A unifying feature of solid tumors is a markedly altered pH profile compared to normal tissues. This reflects that solid tumors, despite completely different origins, often share several phenotypic properties with implications for intra- and extracellular pH. These include: a metabolic shift in most cancer cells toward more acid-producing pathways, reflecting both oncogenic signaling and the development of hypoxia in poorly perfused regions of the tumors; the poorly perfused and often highly dense tumor microenvironment, reducing the diffusive flux of acid equivalents compared to that in normal tissues; and the markedly altered regulation of the expression and activity of pH-regulatory transport proteins in cancer cells. While some of these properties of tumors have been well described in recent years, the great majority of the research in this clinically important area has focused on proton transport, in particular via the Na^+^/H^+^ exchanger 1 (SLC9A1, NHE1) and various H^+^ ATPases. We have, however, recently demonstrated that at least under some conditions, including *in vitro* models of HER2 positive breast cancer, and measurements obtained directly in freshly dissected human mammary carcinomas, bicarbonate transporters such as the electroneutral Na^+^, HCO^−^_3_ cotransporter (SLC4A7, NBCn1), are upregulated and play central roles in pH regulation. In this review, we summarize and discuss the current knowledge regarding the regulation and roles of bicarbonate transporters in cancer. Furthermore, we present new analyses of publicly available expression data demonstrating widely altered expression levels of SLC4- and SLC26 family transporters in breast-, lung-, and colon cancer patients, and we hypothesize that bicarbonate transporter dysregulation may have both diagnostic and therapeutic potential in cancer treatment.

## Introduction and overview

Intracellular pH (pH_i_) homeostasis is a prerequisite for normal cell function and requires, under most conditions, net extrusion of acid equivalents to compensate for the metabolic acid production and the H^+^ influx caused by the inwardly directed driving force for this ion. Within the last decade, numerous studies have demonstrated that pH_i_ homeostasis is often dramatically altered in cancer (for reviews see Gatenby and Gillies, 2008; Webb et al., 2011; Boedtkjer et al., 2012; Parks et al., 2013; Pedersen and Stock, 2013). This has sparked substantial interest in pH regulation as a potential therapeutic target relevant to many forms of cancer. It has been demonstrated that the high metabolic demand of rapidly proliferating cancer cells, in conjunction with a shift toward glycolytic metabolism reflecting both tumor hypoxia and oncogene-induced changes in gene expression, leads to an often greatly increased production of acid equivalents in cancer cells (see Kroemer and Pouyssegur, 2008; Cairns et al., 2011; Cantor and Sabatini, 2012; Andersen et al., 2014). This notwithstanding, cancer cells maintain a pH_i_ that is equal to or even sometimes more alkaline than their normal counterparts, implying that they upregulate net acid extrusion (see Webb et al., 2011; Boedtkjer et al., 2012; Parks et al., 2013; Pedersen and Stock, 2013). This could, in principle, happen by upregulation of the expression and/or activity of acid extruders or, conversely, by downregulation of the expression and/or activity of acid loading transporters—or by some combination thereof, and as will be described below, there is indeed evidence for both in the literature.

Plasma membrane transporters are highly interesting as biomarkers and treatment targets in cancer, given their localization at the cell surface, which renders them easily accessible for e.g., immune-based therapies. Furthermore, acid-base membrane transporters may represent an Achilles' heel for many types of cancers, which due to their high metabolic rates are predicted to be more vulnerable to inhibition of acid extrusion than normal cells. To date, however, only a few acid-base transporters have received attention as potential culprits in the altered acid extrusion pattern in cancer cells. These are the Na^+^/H^+^ exchanger isoform 1, SLC9A1 (NHE1) (e.g., Reshkin et al., 2000; Lauritzen et al., 2010; Boedtkjer et al., 2013), the V-type H^+^ ATPases (Sennoune et al., 2004; You et al., 2009; De Milito et al., 2010), the monocarboxylate transporter family lactate-proton transporters (MCTs, Sonveaux et al., 2008; Halestrap, 2013), and, in a very few cases, HCO^−^_3_-coupled transporters (Ahmed et al., 2009; Lauritzen et al., 2010; Chen et al., 2012; Boedtkjer et al., 2013); see (Parks et al., 2013). With respect to the latter, the best studied in the context of cancer are the electroneutral Na^+^, HCO^−^_3_ cotransporter, SLC4A7 (NBCn1) and the Cl^−^/HCO^−^_3_ exchanger *Down-regulated in Adenoma* (SLC26A3, DRA): NBCn1 is upregulated in breast cancer and single-nucleotide polymorphisms (SNPs) in *SLC4A7* have been linked to increased breast cancer risk while DRA, as the name implies, is essentially lost early upon transformation in many colorectal cancers, and is thought to function as a tumor suppressor.

The aim of this review is to summarize current knowledge on HCO^−^_3_ transport in cancer, and to present and critically discuss evidence regarding the involvement of altered HCO^−^_3_ transporter expression and/or regulation in various cancers. We will first provide an overview of the existing evidence regarding the involvement of the SLC4 (The SLC4 family in cancer—published studies) and SLC26 (The SLC26 family and cancer—published studies) families of HCO^−^_3_ transporters in various cancers. Because of the wealth of information available on these two transporters compared to the rest of the family members, SLC4A7 and SLC26A3 will be discussed separately in the sections SLC4A7 (NBCn1) and Down-Regulated in Adenoma (SLC26A3, DRA), respectively. Finally, in the section Novel Candidates: Other HCO^−^_3_ Transport Proteins with Altered Expression in Cancer, we will explore the expression profiles of the HCO^−^_3_ transporting SLC4 and SLC26 family members in various cancers, using publically available databases, to set the scene for future studies on the regulation and roles of HCO^−^_3_ transport in cancer. Based on the published studies and our new datamining analyses presented here, we hypothesize that HCO^−^_3_ transporter dysregulation may have both diagnostic and therapeutic potential in cancer treatment.

## The SLC4 family in cancer—published studies

**Figure 1:**
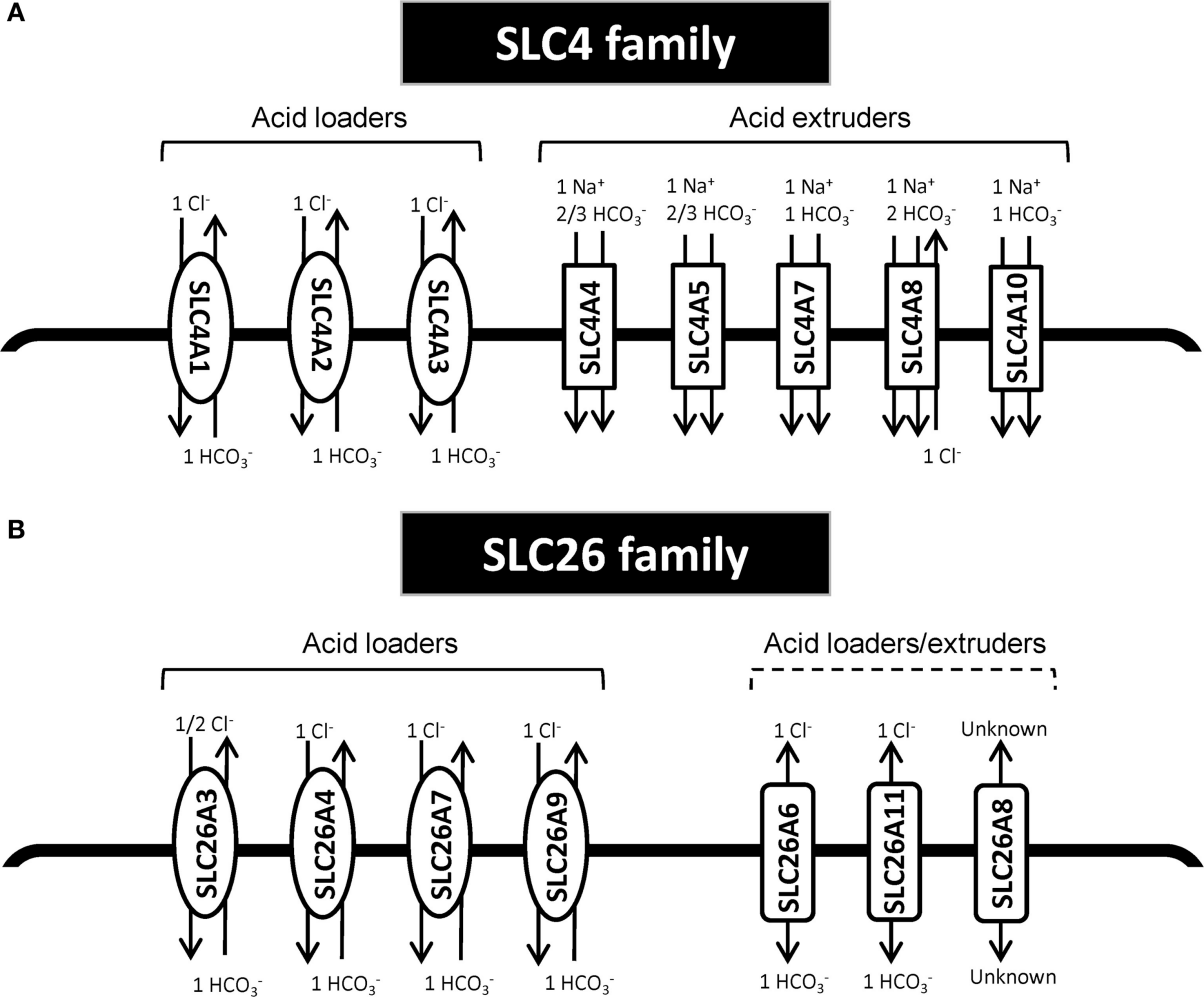
**Schematic model of HCO^−^_3_ transporters from the SLC4 and SLC26 family. (A)** SLC4 family transporters. **(B)** SLC26 family transporters. In each panel, the left side shows the acid loaders and the right side shows the acid extruders. It should be noted SLC4A4 in the kidney and SLC4A5 in the choroid plexus are predicted to have a 1Na^+^:3HCO^−^_3_ stoichiometry and thus would there serve as acid loaders (Millar and Brown, 2008; Romero et al., 2013). Furthermore, SLC4A10 may additionally export Cl^−^; for the differing viewpoints on this, compare (Parker et al., 2008) and (Damkier et al., 2010). Other references: (Yoshitomi et al., 1985; Boron and Boulpaep, 1989; Virkki et al., 2002; Alper and Sharma, 2013; Romero et al., 2013).

The SLC4 family comprises 10 genes, of which at least 8 mediate transport of HCO^−^_3_ (or related molecules, such as CO^2−^_3_) across the plasma membrane (Figure 1). SLC4 family transporters play central roles in pH homeostasis in a wide array of cell types (see Parker and Boron, 2013; Romero et al., 2013). Detailed accounts of the SLC4 family substrates, localization, physiological functions and other pertinent properties can be found elsewhere (see Parker and Boron, 2013; Romero et al., 2013) and will not be provided here. Importantly, however, SLC4A1-3 (frequently denoted anion exchanger (AE) 1–3) are Cl^−^/HCO^−^_3_ exchangers, and at the intra- and extracellular ion concentrations and membrane potential prevalent in most cell types under normal conditions, these proteins will function as cellular acid loaders. Conversely, SLC4A4 (NBCe1), −4A5 (NBCe2), −4A7 (NBCn1), −4A8 (NDCBE), and −4A10 (NCBE/NBCn2) are Na^+^-coupled HCO^−^_3_ transporters, which are predicted to function as cellular acid extruders under the conditions prevalent in most normal cell types. Exceptions are SLC4A4 in the kidney and SLC4A5 in the choroid plexus, which are predicted to have a 1Na^+^:3HCO^−^_3_ stoichiometry and serve as acid loaders (Millar and Brown, 2008; Romero et al., 2013). It may be noted that the sometimes very acidic extracellular pH (pH_*e*_) and consequent low extracellular HCO^−^_3_ concentrations in tumors will diminish the driving force for HCO^−^_3_ import, and could in extreme cases render some of the “acid extruders” net acid importers in the tumor setting. Assuming 150 mM Na^+^_o_, 15 mM Na^+^_i_, and 12 mM HCO^−^_3 *i*_ (pH_*i*_~7.1), the 1Na^+^:1HCO^−^_3_ cotransporters will have an inwardly directed driving force until pH_*e*_ values as low as 6.1, whereas 1Na^+^:2HCO^−^_3_ cotransporters will reverse at much more modest extracellular acidifications.

As noted above, the great majority of studies of pH_i_ dysregulation in cancer have focused on the Na^+^/H^+^ exchanger NHE1, MCT family transporters, and V-type ATPases. Interestingly however, in recent years, several SLC4 family members have been reported to be associated with cancer (Table 1). As mentioned, the most widely studied of these is **SLC4A7** (NBCn1), which has been shown to be upregulated in human breast cancer (Boedtkjer et al., 2013) as well as in cell culture by the ErbB2/HER2 oncogene (Lauritzen et al., 2010, 2012; Gorbatenko et al., 2014), which is upregulated in up to a third of human mammary carcinomas and correlates with poor prognosis, and a SNP in which has been linked to increased risk of breast cancer [see section SLC4A7 (NBCn1), Ahmed et al., 2009; Long et al., 2010; Han et al., 2011; Sueta et al., 2012].

**Table 1:** **Summary of the reported data on HCO^−^_3_ transporting SLC4 and SLC26 family transporters in cancer**.

**Transporter**	**Alternative name**	**Cancer**	**Perturbation**	**Functional link to cancer?**	**References**
SLC4A1	EPB3, AE1	Gastric, CRC	Expression ↑↓	Correlates with cancer progression	Shen et al., 2007; Xu et al., 2009; Wu et al., 2010; Suo et al., 2012
SLC4A2	EPB3L1, AE2	Gastric, CRC, HCC, bladder	Expression ↓↑ SNP rs13240966	Poor prognosis, SNP—reduced risk of bladder cancer	Wu et al., 2006; Yang et al., 2008; Andrew et al., 2009; Hwang et al., 2009; Tian et al., 2010; Song et al., 2012; Belickova et al., 2013; Wang et al., 2013
SLC4A4	NBCe1	PTC, CML, CRC	Expression ↓↑	Resistance to methotrexate	Galeza-Kulik et al., 2006; Kim et al., 2010; Mencia et al., 2011; Gerber et al., 2013
SLC4A7	NBCn1	Breast cancer	Expression (↓)↑ SNP rs4973768	Unknown, SNP—associated with breast cancer risk	Chen et al., 2007; Ahmed et al., 2009; Lauritzen et al., 2010, 2012; Long et al., 2010; Han et al., 2011; Mulligan et al., 2011; Boedtkjer et al., 2012, 2013; Fasching et al., 2012; Sueta et al., 2012; Fernandez-Navarro et al., 2013; Gorbatenko et al., 2014;
SLC26A2	DTDST	CRC	Expression ↓	Reduced sulfation of sialyl Lewis x ligand	Yusa et al., 2010
SLC26A3	DRA, CLD	CRC, Instestina, breast cancer	Expression ↓ Mutations	Reduces proliferation	Schweinfest et al., 1993; Hoglund et al., 1996; Antalis et al., 1998; Hemminki et al., 1998; Chapman et al., 2002; Schweinfest et al., 2006; Lauriola et al., 2010; Baker et al., 2011; Dalerba et al., 2011; Mosakhani et al., 2012; de Ronde et al., 2013
SLC26A4	PDS, pendrin	Thyroid cancer	Expression ↓	Unknown	Arturi et al., 2001; Porra et al., 2002; Xing et al., 2003; Iwata et al., 2011

Arrows (↑↓) indicate reported up- or down-regulation, respectively. See text for details. Abbreviations: EPB3, Erythrocyte Membrane Protein Band 3; AE1, Anion Exchanger 1; EPB3L1, Erythrocyte Membrane Protein Band 3 Like 1; AE2, Anion Exchanger 2; NBCe1, Na^+^, HCO*_3_*^−^ electrogenic co-transporter 1; NBCn1, Na^+^, HCO*_3_*^−^ neutral co-transporter 1; DTDST, Diastrophic Dysplasia Sulfate Transporter; DRA, Down-Regulated in Adenoma; CLD, Congenital Chloride Diarrhoea; PDS, Pendred Syndrome.

Dysregulation of several other members of the SLC4 family has been linked to cancer development (Table 1). An interesting series of studies relate the Cl^−^/HCO^−^_3_ exchangers SLC4A1 (AE1) and SLC4A2 (AE2) to gastrointestinal cancers. **SLC4A1**, which is normally only expressed at detectable protein levels in erythrocytes, was found to be markedly upregulated in gastric cancer cells and colorectal cancer (CRC) cells (Shen et al., 2007), and its expression to correlate with cancer progression (Xu et al., 2009). Interestingly, SLC4A1 was not found in the plasma membrane, but exclusively in the cytoplasm of these cells (Shen et al., 2007). The tumorigenic effect of SLC4A1 was proposed to involve a direct interaction of its C-terminal with the tumor suppressor p16 (INK4a), sequestering the latter from the nucleus, where it normally functions to inhibit cell cycle progression (Shen et al., 2007). Thus, SLC4A1 was proposed to increase gastric carcinogenesis by favoring cell proliferation, in a manner apparently independent of effects on pH_i_ regulation (Shen et al., 2007). Knockdown of SLC4A1 reduced tumor progression in two different mouse models of gastric cancer, a xenograft model and chemical induction in conjunction with *H. pylori* (Suo et al., 2012). Interestingly, the upregulation of SLC4A1 in gastric cancer was recently found to be dependent on aberrant regulation by miR-24, which normally silences SLC4A1 protein expression by interaction with the 3′ untranslated region (UTR) of the gene (Wu et al., 2010).

**SLC4A2** (AE2) was found to be upregulated in CRC (Song et al., 2012) and hepatocellular carcinoma (HCC) (Wu et al., 2006; Hwang et al., 2009) and similar to SLC4A1 appears to interact with p16 in the cytosol (Song et al., 2012). In CRC, AE2 expression correlated with Ki67 proliferation marker staining and with poor prognosis (Song et al., 2012). In gastric cancer, both upregulation (Wang et al., 2013) and downregulation (Yang et al., 2008) of SLC4A2 has been reported, and this was proposed to reflect that its expression is regulated by gastrin, the expression of which varies between different gastric cancers and cancer stages (Wang et al., 2013). Gastrin treatment decreased pH_i_ of human gastric cancer cells in a manner correlating with the increased expression of SLC4A2 (Wang et al., 2013). On the other hand, in CRC, gastrin treatment appeared to decrease SLC4A2 expression, by interfering with binding of EGR1 to the *AE2a1* promoter (Song et al., 2012; Wang et al., 2013). Furthermore, gastrin was found to reduce SLC4A1 expression in gastric cancer cells by eliciting its protein degradation (Tian et al., 2010). Thus, the precise regulation of SLC4A1 and -A2 in gastrointestinal cancers requires further elucidation and may be cell type specific. Moreover, whereas in contrast to SLC4A1, SLC4A2 does to some extent localize to the plasma membrane in the cancer cells (Song et al., 2012; Wang et al., 2013), it is not clear from the published studies whether the role of the exchanger in cancer progression involves both pH_i_ regulation and p16 sequestration.

A SNP, rs13240966, located to an intronic region of the *SLC4A2* gene, was found to be associated with *reduced* risk of bladder cancer, with an odds ratio for homozygotes of 0.6 (Andrew et al., 2009). On the other hand, this same SNP significantly associated with susceptibility to myelodysplastic syndromes—hematopoetic diseases that frequently become acute leukemias—in a cohort of Czech patients (Belickova et al., 2013). Impressively, homozygosity for this SNP was associated with an odds ratio of 4.86 for myelodysplastic syndromes (Belickova et al., 2013). It is to our knowledge unknown whether this intronic SNP alters the protein expression pattern of SLC4A2, and the causal link, if any, between SLC4A2 and myelodysplastic syndromes still needs to be established. It is, however, well known that transcriptional regulatory elements may be located in introns, and regulation of ion transporter expression by an intronic SNP has previously been reported (Tokuhiro et al., 2003).

Two studies of papillary thyroid cancers (PTC) in patients of Polish (Galeza-Kulik et al., 2006) and Korean (Kim et al., 2010) descent showed that mRNA expression of **SLC4A4** (NBCe1) was decreased by 7- and 3-fold, respectively, in PTC compared to normal tissue. In contrast, a more recent study found SLC4A4 mRNA levels to be significantly (2.5 fold) upregulated in chronic myeloid leukemia (CML) stem cells compared to normal stem and progenitor cells, and was proposed as a possible target for immune-based therapy due to its cell surface localization (Gerber et al., 2013). Neither the mechanism(s) of SLC4A4 dysregulation nor the possible causal role in CML were, however, addressed in these studies. Interestingly, in a study in HT29 human CRC cells, expression of SLC4A4 was shown to be negatively regulated by miR-224 (Mencia et al., 2011). In HT29 cells with acquired resistance to methotrexate (a folate- and metabolism-targeting agent) chemotherapy, miR-224 was downregulated, resulting in upregulation of SLC4A4. Notably, siRNA-mediated knockdown of SLC4A4 resulted in increased sensitivity to methotrexate treatment, and this was reversed upon anti-miR-224 transfection (Mencia et al., 2011).

## The SLC26 family and cancer—published studies

The second major group of mammalian plasma membrane HCO^−^_3_ transporters is the SLC26 family. This family comprises 11 genes of which 8 have been shown to carry HCO^−^_3_ (see Alper and Sharma, 2013). All of the SLC26 members function as anion exchangers, and although the stoichiometry for these transporters is not fully elucidated, they appear to most commonly serve as cellular acid loaders (Figure 1). Three of them—SLC26A7, −9, and −11 can additionally operate as anion channels (see Alper and Sharma, 2013). Dysregulation of several members of this family has been linked to cancer development (Table 1). As noted above, one in particular has received widespread attention for its dysregulation in CRC, namely **SLC26A3**, also known as *Downregulated in Adenoma* (DRA), which was first described as a gene downregulated in CRC in 1993 (Schweinfest et al., 1993). The extensive body of evidence on the regulation and roles of SLC26A3 in cancer is discussed in the section Down-Regulated in Adenoma (SLC26A3, DRA).

A few other SLC26 family members have been linked to cancer. Expression of **SLC26A2** (DTDST), an exchanger carrying Cl^−^, oxalate and SO^2−^_4_, but not thought to transport HCO^−^_3_ (see Alper and Sharma, 2013), was found to be strongly reduced in CRC, likely due to epigenetic silencing (aberrant histone methylation and acetylation) (Yusa et al., 2010). This was assigned a role in reducing sulfation of sialyl Lewis^*x*^ and increasing proliferation in CRC (Yusa et al., 2010). **SLC26A4** (Pendrin) exhibits reduced expression levels in thyroid cancers (Arturi et al., 2001; Porra et al., 2002). The possible causal role of this downregulation in cancer development is to our knowledge unknown, but given the role of SLC26A4 as an anion exchanger capable of transporting Cl^−^, HCO^−^_3_, and I^−^, it is tempting to speculate that its downregulation may contribute to maintaining an elevated pH_i_ in these cells. A role for I^−^ transport is of course also conceivable, however, the SLC26A4 knockout mouse has no thyroid phenotype even under dietary iodide deficiency (Iwata et al., 2011), suggesting that this transporter is not the main I^−^ uptake pathway in the thyroid. Interestingly, the 5′ region of the *SLC26A4* gene was found to be hyper-methylated as an early event in the majority of malignant human thyroid tumors, presumably accounting for at least part of the decreased SLC26A4 expression in these tumors (Xing et al., 2003).

## Function and expression of HCO^−^_3_ transporters in cancer

### Roles of the carbonic buffer system in tumor pH regulation

Recent studies from several laboratories have documented the specific importance of HCO^−^_3_ in tumor pH regulation. Intriguingly, it was demonstrated that oral administration of HCO^−^_3_ could inhibit tumor metastasis in prostate-, breast- and colon cancer cell line derived tumors. This apparently occurred by reducing the extracellular acidity of the highly acid-extruding tumors, in absence of changes in the pH of blood or normal tissues (Robey et al., 2009; Silva et al., 2009; Wojtkowiak et al., 2011; Estrella et al., 2013). Furthermore, early administration of HCO^−^_3_ prevented carcinogenesis in *Transgenic Adenocarcinoma of the Mouse Prostate* (TRAMP) mice, a model of prostate cancer (Ibrahim-Hashim et al., 2012). Although the mechanisms underlying these effects are not absolutely clear, the authors suggest that increased tumor pH_*e*_ inhibits the activity of extracellular proteases, such as Cathepsin B, in turn reducing invasiveness (Robey et al., 2009). This hypothesis was recently supported by the finding that *in vivo* matrix metalloproteinase and cathepsin activities, estimated by fluorescent reporters, are reduced in xenograft tumors following NaHCO_3_ ingestion (Robey and Nesbit, 2013). A recent study, however, points to the drawbacks and the possible side effects of chronic HCO^−^_3_ administration such as metabolic alkalosis and additionally suggests combinational therapy targeting proton production with dichloroacetate (DCA) (Martin et al., 2012).

The importance of the carbonic buffer system in cancer is also underscored by the ability of the hypoxia inducible carbonic anhydrase IX (CAIX), which is upregulated in a wide variety of cancers (Bartosova et al., 2002; Chen et al., 2005; Supuran, 2008; Perez-Sayans et al., 2012), to increase HCO^−^_3_-dependent resting pH_i_ and decrease pH_*e*_ in Chinese hamster lung CCL39 fibroblasts. CAIX knockdown has also been shown to reduce growth of xenografts of LS174Tr colon cancer cells in nude mice (Chiche et al., 2009). While most of the work on carbonic anhydrases in cancer has focused on CAIX as a biomarker, studies from several groups have highlighted the importance of the carbonic buffer system for pH regulation in various cancer cell lines under normal, acidic, and hypoxic growth conditions When cancer cell lines from pancreas, breast, and colon were grown in 2D culture, the mechanism of net acid extrusion after an imposed acid load varied between cell lines, from mainly HCO^−^_3_ influx to mainly H^+^ efflux (Hulikova et al., 2011). Notably, at a pH_*e*_ of 7.4, the HCO^−^_3_ flux was rather similar between cell lines, whereas the H^+^ efflux varied substantially (Hulikova et al., 2011). The ability to recover from an imposed acid load was reduced by decreasing pH_*e*_. This decrease could be mostly ascribed to reduced H^+^ efflux, whereas the HCO^−^_3_ influx was less affected by acidic pH_*e*_ (Hulikova et al., 2011). A similar pattern was observed after exposing cancer cell lines to hypoxia, which reduced SLC9A1-mediated H^+^ flux, whereas HCO^−^_3_ flux was hypoxia-insensitive (Hulikova et al., 2013). Collectively, these studies suggest that HCO^−^_3_ transport may serve a rather stable “housekeeping” role in these cell lines, whereas H^+^ transport is highly variable and attenuated under conditions of extracellular acidification and hypoxia. As these conditions are prevalent in solid tumors (Vaupel et al., 1981; Vaupel, 2004), the authors next addressed the mechanisms of pH_i_ regulation in multicellular spheroids mimicking the tumor 3D structure. These studies demonstrated that inhibition of HCO^−^_3_ transport by 4,4′-diisothiocyanatostilbene-2,2′-disulfonic acid (DIDS) generally lead to a non-uniform decrease in the rate of pH_i_ recovery after acid loading, with the greatest effect of DIDS observed in the core of spheroids. Importantly, the authors provide evidence that the carbonic buffer system plays a dual role, supplying HCO^−^_3_ as substrate for transport and providing a mobile buffer facilitating the transport of the otherwise poorly mobile H^+^ ions away from the cells. Thus, in MDA-MB-468 breast cancer spheroids, which rely mainly on H^+^ transport for pH_i_ recovery, substitution of HCO^−^_3_ with 25 mM HEPES had no effect on the ability to recover from an acid load. However, decreasing the concentration of HEPES elicited a non-uniform pattern of pH_i_ recovery with the slowest recovery at the core of the spheroid consistent with CO_2_/HCO^−^_3_ (and under experimental conditions HEPES) serving as a mobile buffer important for pH_i_ recovery under 3D conditions (Hulikova et al., 2011). Moreover, the findings predict that because of the inhibitory effects of extracellular acidification and hypoxia on SLC9A1, HCO^−^_3_ transport will be progressively more important with distance from functional blood vessels in the tumor. Finally, inhibition of either SLC9A1 or HCO^−^_3_ transport attenuated spheroid growth, suggesting that both types of transporters are important for cancer cell survival and/or proliferation under 3D conditions (Hulikova et al., 2011).

As mentioned above, the strongest evidence for involvement of HCO^−^_3_ transporters in human cancer has been provided for NBCn1 (SLC4A7) and DRA (SLC26A3). The current evidence linking these two transport proteins to cancer will be addressed below.

### SLC4A7 (NBCn1)

The first link between this protein and breast cancer was provided when SLC4A7 was reported to be a tyrosine kinase substrate in MCF10AT breast cancer cells and to be expressed at reduced levels in human breast cancer samples compared to matched normal breast tissue in a population of Asian women (Chen et al., 2007). However, in the MCF10AT progression model, SLC4A7 expression was approximately three times higher in low- and intermediate-grade lesions than in normal cells (Chen et al., 2007) suggesting that at least early in breast cancer development, SLC4A7 expression may be upregulated. The potential importance of these observations was supported when multiple genome-wide association studies (GWAS) subsequently showed that a SNP in the *SLC4A7* gene was linked to breast cancer: independent GWAS covering women of European (Ahmed et al., 2009), Korean (Han et al., 2011), Chinese (Long et al., 2010), and Japanese (Sueta et al., 2012) descent showed that the rs4973768 SNP corresponding to the 3′-UTR of *SLC4A7* is associated with breast cancer with odds ratios for homozygosity between 1.2 and 1.3. The rs4973768 SNP has an allele frequency between 15 and 50% across the range of investigated ethnicities (Ahmed et al., 2009; Long et al., 2010; Han et al., 2011; Sueta et al., 2012) and thus could play a substantial role for human breast cancer development. In carriers of the cancer susceptibility gene BRCA2, the SNP was significantly associated with estrogen receptor (ER)-positive, but not with ER-negative disease (Mulligan et al., 2011). This suggests a possible link to ER signaling, which may also underlie the apparent dependence of the breast cancer association of this SNP on pre- or post-menopausal status (Chen et al., 2012; Fernandez-Navarro et al., 2013).

Although it has been noted that the rs4973768 SNP may alter the binding affinity of a microRNA binding site (Boedtkjer et al., 2012) and thus alter *SLC4A7* mRNA stability and translation, the consequences of the rs4973768 SNP for SLC4A7 expression and function have not yet been experimentally determined. Of note, the rs4973768 SNP did not link to breast cancer survival in a large GWAS study of 25853 breast cancer patients with available follow-up data (Fasching et al., 2012). Moreover, there was no association between rs4973768 and mammographic density—a risk factor for breast cancer—except in a subgroup of pre-menopausal women (Fernandez-Navarro et al., 2013).

Human breast cancer is a fairly heterogeneous disease, which differs among others in expression levels for hormone (e.g., estrogen and progesterone) and growth factor (e.g., ErbB2) receptors. The receptor status of the breast carcinomas modifies prognosis. In particular, expression of a constitutively active, NH_2_-truncated ErbB2 (ΔNErbB2) receptor has been widely shown to confer poor prognosis and limit treatment options (Christianson et al., 1998). In this light, it is interesting that heterologous expression of the Δ NErbB2 receptor in human MCF-7 breast cancer cells enhances expression of SLC4A7 and cellular acid extrusion activity (Lauritzen et al., 2010).

**Figure 2:**
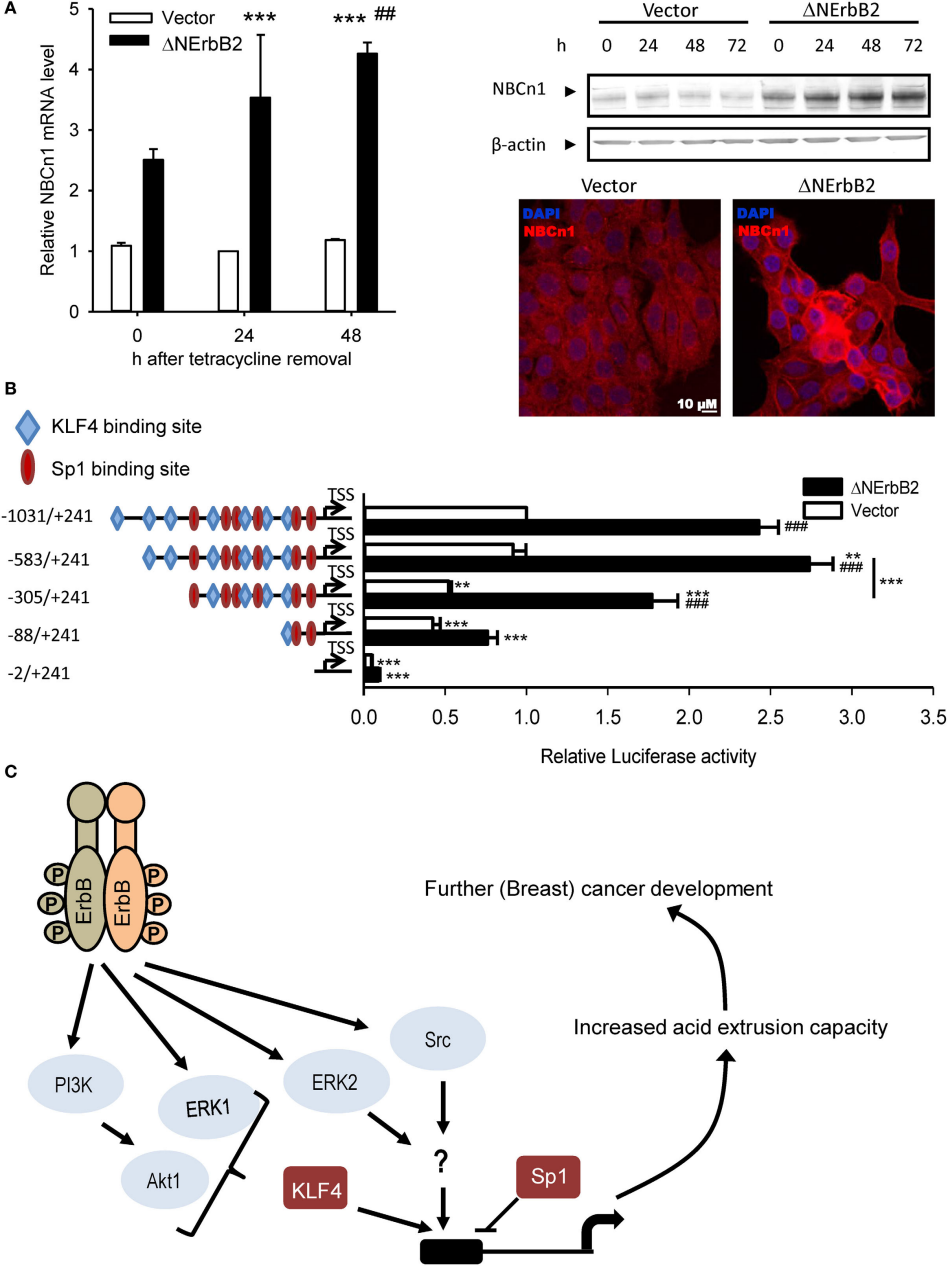
**SLC4A7 expression regulation by the truncated ErbB2 receptor**. **(A)** SLC4A7 mRNA and protein levels. MCF-7 cells with a stably cloned N-terminally truncated ErbB2 receptor under control of a tetracycline-off promoter (MCF-tTA-ΔNErbB2; here referred to as ΔNErbB2 cells) and corresponding vector controls (MCF-tTA-pTRE; referred to as vector cells) were used. At given time points, after tetracycline removal, cells were lysed or paraformaldehyde fixed. Left: mRNA levels were assessed using SYBRGreen qPCR. ^***^P < 0.001 compared to vector cells at 24 h. ^##^ Indicate *P* < 0.05 and *P* < 0.01, respectively, compared to the corresponding time point in vector cells. Right: protein levels, representative Western blot. Below: Representative immunofluorescence image. **(B)** *SLC4A7* promoter activity and regulation by ErbB2. Cells were co-transfected with the pGL3 basic vector, in which the relevant DNA constructs were cloned upstream of the firefly luciferase gene. Promoter activity was assessed by dual luciferase assays. At 24 h after tetracycline removal to induce ΔNErbB2 expression, cells were transfected with the indicated constructs and with the pHRG-B vector carrying *Renilla* luciferase, and luciferase activity was measured another 24 h later. Data are shown as firefly luciferase activity normalized to *Renilla* luciferase activity, relative to that in vector cells. Numbers indicate sequence length; − and + indicate sequence position up- or down-stream, respectively, of the TSS obtained from CAGE data. Blue rhombuses and red circles indicate binding sites for KLF4 and Sp1, respectively. Only binding sites with relative profile score threshold >90% of Jaspar position weight matrices (PWMs) are depicted. Data are from 3 to 6 independent experiments/conditions. ^**^P < 0.01, ^***^P < 0.001 vs. full-length sequence in same cell type unless indicated with a line; ^###^*P* < 0.001 vs. other cell type in same conditions. **(C)** Model of ErbB2 dependent SLC4A7 expression regulation. See text for details. Data in **(A)** are from Lauritzen et al. (2010), except immunofluorescence data, which are unpublished data by C.W. Olesen. Data in **(B)** and the model in **(C)** are redrawn with permission, from Gorbatenko et al. (2014).

Recent studies from our group demonstrated that Δ NErbB2-dependent SLC4A7 upregulation is controlled via Akt-, ERK- and Src-dependent pathways (Gorbatenko et al., 2014; Figure 2). Inhibition or siRNA-mediated knockdown of these kinases decreased *SLC4A7* mRNA and protein levels. Furthermore, the *SLC4A7* promoter was characterized in detail. Although *SLC4A7* was suggested to have several alternative promoters, all epigenetic marks, correlating with presence of actively transcribed chromatin, such as H3K27Ac and H3K27me3, were only detectible near the first possible *SLC4A7* exon. The *SLC4A7* core promoter is TATA-box-less, yet contains a large GC rich region. Despite the fact that CpG island containing promoters usually exhibit broad transcription initiation, Cap Analysis of Gene Expression (CAGE) data from MCF-7 breast cancer cells suggested a single base-focused transcription start site (TSS) and the sequence around it perfectly resembled an initiator (Inr) element (Gorbatenko et al., 2014). Luciferase reporter assays of the putative *SLC4A7* promoter region demonstrated that mutation of the Inr element led to 50% loss of promoter activity (Figure 2). Δ NErbB2 signaling stimulated the promoter activity by ~2.5 fold and further deletions identified the minimal, Δ NErbB2 sensitive region within the promoter. Two related transcription factors with known roles in cancer, Sp1 and KLF4, bind to the *SLC4A7* promoter, exerting opposite functions: Sp1 represses and KLF4 activates *SLC4A7* transcription (Gorbatenko et al., 2014). Notably, KLF4-dependent transcription was stimulated by Δ NErbB2 signaling and this was at least partially controlled by Akt1 and ERK1 kinases (Figure 2). Finally, stimulation of full-length ErbB2 receptors in SKBr3 breast cancer cells increased both SLC4A7 expression and HCO^−^_3_-dependent pH_*i*_ recovery (Gorbatenko et al., 2014).

Although the initial investigations in a population of Asian women suggested that the expression levels for SLC4A7 are reduced in breast cancer tissue compared to normal breast tissue (Chen et al., 2007), this has not subsequently been confirmed. In contrast, based on tissue samples from women of European descent, we recently showed that the plasma membrane expression of SLC4A7 is upregulated in human primary breast carcinomas and metastases compared to matched normal breast tissue (Boedtkjer et al., 2013). We furthermore showed that Na^+^, HCO^−^_3_ cotransport of low sensitivity to DIDS, a hall-mark of epithelial SLC4A7-mediated HCO^−^_3_ transport, (Parker and Boron, 2013) is the predominant mechanism of acid extrusion in freshly isolated slices of human primary breast carcinomas (Boedtkjer et al., 2013). Interestingly, only 10–20% of the investigated human breast carcinomas in our study showed upregulation or gene amplification of the ErbB2 receptor, suggesting that upregulated SLC4A7 expression is of general importance in breast cancer and not restricted to cancers characterized by increased ErbB2 signaling (Boedtkjer et al., 2013). The enhanced expression of SLC4A7 and its putative role in cellular acid extrusion would imply that SLC4A7 is involved in establishing the characteristically high pH_i_ and low pH_*e*_ observed in breast carcinomas. In congruence, steady-state pH_i_ in slices of human primary breast carcinomas decreased around 0.35 units upon omission of CO_2_/HCO^−^_3_ (Boedtkjer et al., 2013).

### Relative roles of SLC4A7 (NBCn1) and SLC9A1 (NHE1) in cancer

In most cell systems, Na^+^, HCO^−^_3_ cotransport acts in parallel with Na^+^/H^+^ exchange to extrude intracellular acid loads, and a brief discussion of their relative roles in cancer is therefore warranted. Multiple studies have demonstrated that Na^+^/H^+^ exchange plays a significant role in cancer cells (Reshkin et al., 2013) and xenograft studies have shown that Na^+^/H^+^ exchange deficiency inhibits tumor growth of MGH-U1 bladder carcinoma cells (Rotin et al., 1989). SLC9A1 has been shown to contribute to pH_i_ regulation in cultured MCF-7 cells (Lauritzen et al., 2010) and human breast cancer slices (Boedtkjer et al., 2013). In MCF-7 cells, no significant change in SLC9A1 expression was observed in response to heterologous expression of Δ NErbB2, yet the contribution of SLC9A1 to cellular acid extrusion was substantial (Lauritzen et al., 2010) and Δ NErbB2 expression was associated with increased phosphorylation of SLC9A1 at Ser703 (Lauritzen et al., 2012), which has previously been shown to increase SLC9A1 activity (Takahashi et al., 1999). In the human breast cancer slices, Na^+^/H^+^ exchange contributed to net acid extrusion primarily at low pH_i_ values (Boedtkjer et al., 2013). Although the role of Na^+^/H^+^ exchange may be less obvious than the role of Na^+^, HCO^−^_3_ cotransport for cellular net acid extrusion and global steady-state pH_i_ control in breast carcinomas (Boedtkjer et al., 2013), cell culture experiments have clearly demonstrated that SLC9A1 plays a prominent and specific role for modulating cell migration, survival, growth and proliferation (Stock and Schwab, 2006; Schwab et al., 2012). These effects may in part be explained by changes in pH in local restricted compartments (Ro and Carson, 2004) or may be due to transport-independent interactions with intra- or extracellular structural components or with cellular signaling cascades (Stock and Schwab, 2006). Initial studies to explore whether SLC4A7 plays a similar role for cell migration have been conducted using pharmacological inhibitors of Na^+^/H^+^ exchange (5-(N-ethyl-N-isopropyl) amiloride, EIPA) and Na^+^, HCO^−^_3_ cotransport (S0859) in Δ NErbB2-expressing MCF-7 breast cancer cells: EIPA was surprisingly shown to enhance cell migration while S0859 had no effect (Lauritzen et al., 2012). An earlier study suggested that the electrogenic Na^+^, HCO^−^_3_ cotransporter NBCe1 had a minor stimulatory role on migration in transformed Na^+^/H^+^ exchange deficient Madin-Darby Canine Kidney (MDCK) cells (Schwab et al., 2005), providing evidence that Na^+^, HCO^−^_3_ cotransport can modulate cell motility at least in some cell systems.

Since SLC4A7 is also expressed in the smooth muscle and endothelial cells of the vascular wall (Boedtkjer et al., 2008) and is important for maintaining vasomotor responsiveness (Boedtkjer et al., 2011) and possibly arterial structure (Boedtkjer and Aalkjaer, 2013), it is conceivable that SLC4A7 may also in part affect cancer development by modifying blood perfusion of tumors. Efficient acid extrusion mechanisms in the vascular smooth muscle cells and endothelial cells of tumor vessels would be expected to be particularly important due to the high local acid load.

### Down-regulated in adenoma (SLC26A3, DRA)

SLC26A3 was first described based on its reduced expression levels in colorectal adenomas and adenocarcinomas compared to normal colon epithelium (Schweinfest et al., 1993). Subsequently, SLC26A3 was reported to function as a Cl^−^/anion exchanger in the luminal membrane of the gastrointestinal tract and was proposed to contribute to transepithelial HCO^−^_3_ secretion (Jacob et al., 2002). The anions transported by SLC26A3 appear to include Cl^−^, HCO^−^_3_ and SO^2−^_4_ while transport of oxalate is limited (Jacob et al., 2002). Since SLC26A3 can remove HCO^−^_3_ from cells, it is possible that lower expression of SLC26A3 reduces the net acid load on the cells and eases the elimination of acidic waste products produced by cancer cell metabolism. The role of SLC26A3 for pH_i_ regulation in colon carcinomas, however, needs further investigation.

Mutation of SLC26A3—in addition to causing congenital chloride diarrhea (Hoglund et al., 1996)—increases the risk of intestinal cancer (Hemminki et al., 1998). It is not clear at this point, however, whether the correlation between SLC26A3 and colon cancer is due to its role in HCO^−^_3_ transport (and hence to regulation of pH_i_ and local pH_*e*_), a role in cellular Cl^−^ homeostasis, or other less understood functions. Interestingly, mutations in the Cl^−^ channel Cystic Fibrosis Transmembrane conductance Regulator (CFTR), which is expressed in the luminal membrane of colonic crypt cells (Crawford et al., 1991), have also been suggested to be inversely associated with colon cancer (Padua et al., 1997), supporting the possibility for Cl^−^-dependent effects of SLC26A3. SLC26A3 has, furthermore, been proposed to modify cellular growth: heterologous expression of SLC26A3 induces growth suppression in various cancer cell lines (Chapman et al., 2002) while SLC26A3 knockout mice display altered morphology of the colonic mucosa with an expanded proliferative zone (Schweinfest et al., 2006). In combination, these findings suggest multiple possible roles of SLC26A3 in cancer development, and additional experimental investigations are required to further our mechanistic understanding. Notably, however, SLC26A3 expression, together with expression of three other genes, had predictive value for survival in patients with CRC of the wild type KRAS type (Baker et al., 2011).

SLC26A3 downregulation correlates with colon tumor progression (Antalis et al., 1998); and in congruence with this, expression levels for SLC26A3 have been reported to be controlled by miR-31, which is upregulated in tumors from patients with progressive CRC compared to patients with disease control (Mosakhani et al., 2012). Although SLC26A3 has been shown to be a prognostic marker in colon cancer (Dalerba et al., 2011), a potential causative role of SLC26A3 expression in colon carcinogenesis is still controversial: some investigators have shown that SLC26A3 expression correlates with cell differentiation, i.e., expression of SLC26A3 occurs preferentially in highly differentiated colonic epithelial cells but is low in dedifferentiated cells (Hoglund et al., 1996). The association between SLC26A3 expression levels and cellular differentiation in the normal epithelium, however, does not appear to be recapitulated in tumors since SLC26A3 expression has been shown to be reduced in both differentiated and dedifferentiated tumors (Antalis et al., 1998). Since cancer cells are generally dedifferentiated compared to normal epithelial cells, however, it cannot currently be excluded that reduced expression of SLC26A3 in colorectal adenocarcinomas is a consequence of the dedifferentiated state rather than a mechanistic cause of malignant development.

Since SLC26A3 is expressed in colonic epithelium but not in blood cells, detection of circulating tumor cells by identification of SLC26A3 mRNA was suggested as a screening method for CRC, but the specificity has so far been too low for SLC26A3 to qualify as a clinically useful marker (Lauriola et al., 2010).

It is currently unclear from the literature whether SLC26A3 is also of relevance to other types of cancer (see, however, the section Novel Candidates: Other HCO^−^_3_ Transport Proteins with Altered Expression in Cancer); but consistent with more general implications, SLC26A3 was recently shown to be a marker of resistance to neoadjuvant chemotherapy in ErbB2 receptor negative breast cancer (de Ronde et al., 2013).

## Novel candidates: other HCO^−^_3_ transport proteins with altered expression in cancer

In the previous sections, we have discussed the published literature on HCO^−^_3_ transporters in relation to cancer. As evident from this, only a few studies directly addressing this issue are yet available, and for most HCO^−^_3_ transporters, their possible relation to cancer development is completely uninvestigated. In order to identify novel potential candidates for dysregulation of HCO^−^_3_ transport in cancer, we have mined two authoritative bioinformatics sources: The Catalog of Somatic Mutations in Cancer (COSMIC) database of cancer-associated mutations (http://cancer.sanger.ac.uk, Forbes et al., 2011) and The Cancer Genome Atlas (TCGA, http://cancergenome.nih.gov/), the latter using the Cancer Genomics Browser (https://genome-cancer.ucsc.edu/, Zhu et al., 2009; Goldman et al., 2013).

Table 2 summarizes mutation rates for SLC4 and SLC26 members extracted from COSMIC. As seen, the mutation rates for the SLC4 and SLC26 families vary markedly between the five different cancer types shown. Less than 1% of the genes from these two families exhibit somatic mutations in breast and pancreatic cancer. In contrast, in endometrial cancers, the mutation rate is 5% or higher for almost all of the SLC4 and SLC26 genes. This should be seen in the light of the generally high frequency of genomic instability in endometrial cancers (Matias-Guiu and Prat, 2013), however, it would be highly interesting to assess whether any of these mutations alter transporter function. In colon and lung cancer, the mutation rates are very different between the different genes, but it is interesting to note that overall, the SLC4 family has a higher mutation rate compared to the SLC26 family in these two cancer types. These data, while preliminary, suggest that mutations in HCO^−^_3_ transporters could be present in multiple cancers and it would be interesting to address in future studies whether specific mutations with effects on transporter function might contribute to the cancer phenotype.

**Table 2:**
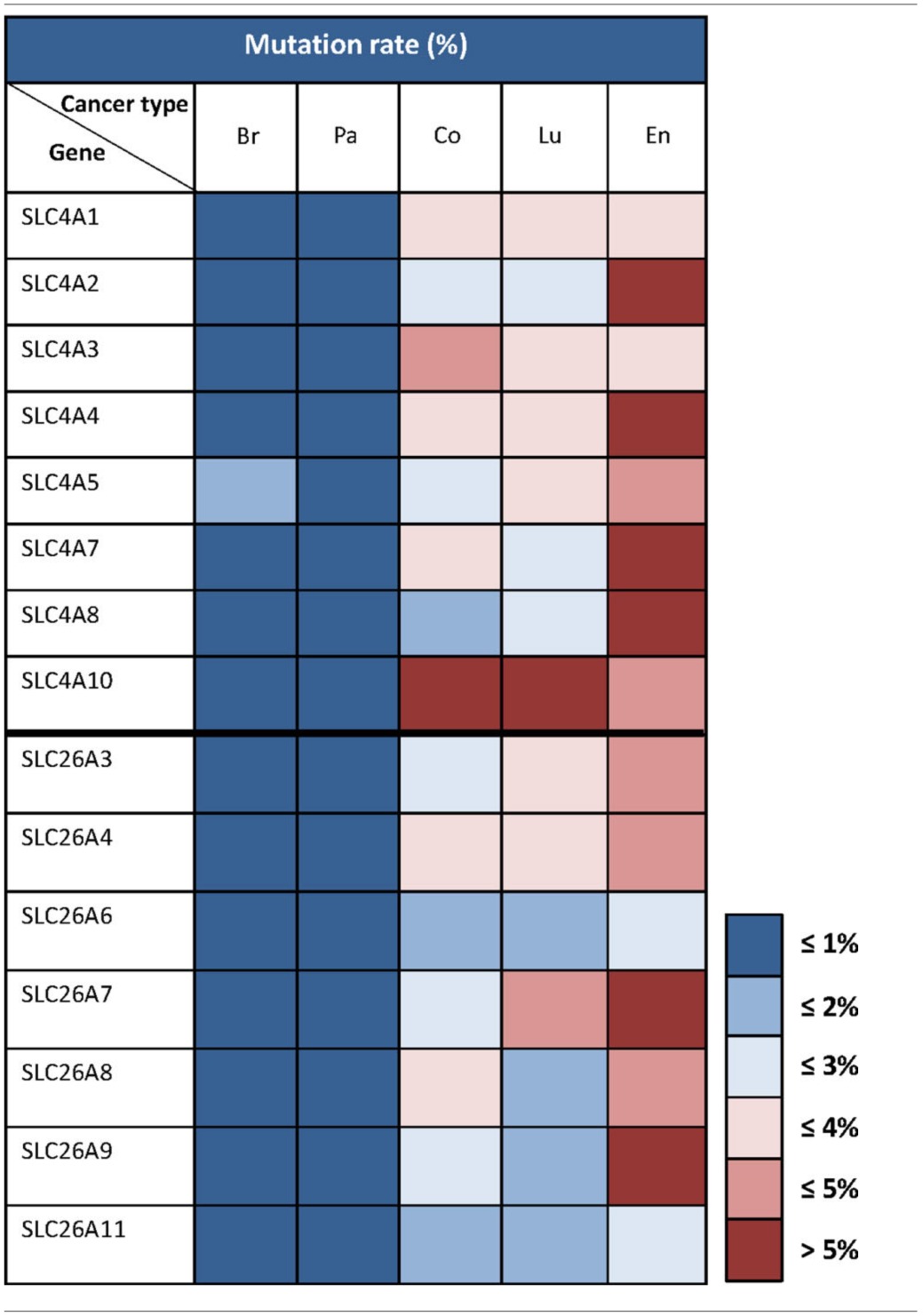
**Somatic mutation rates for SLC4 and SLC26 family transporters in different cancer types**.

Data are obtained from the COSMIC cancer mutation database. See text for details.

**Figure 3:**
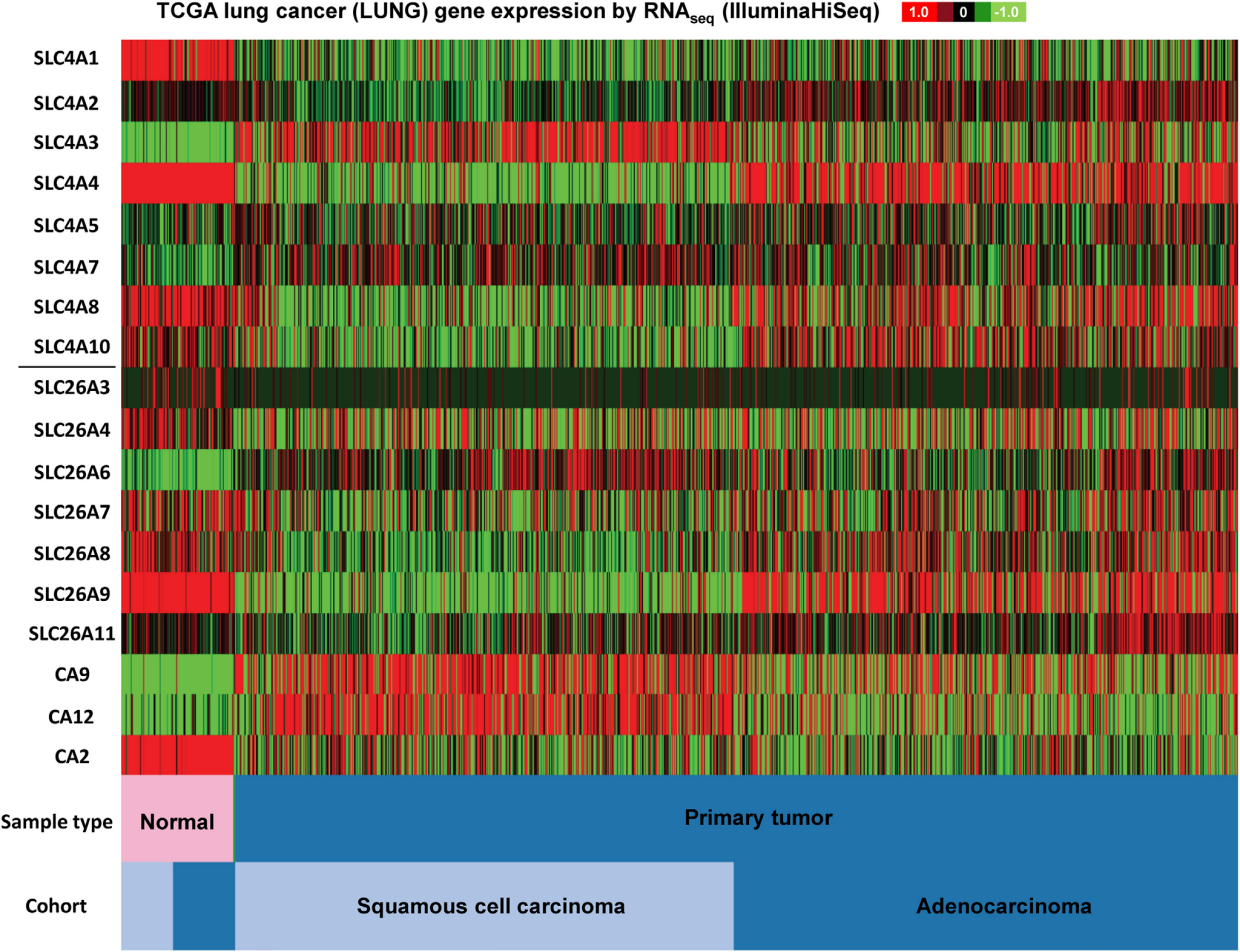
**SLC4 and SLC26 family mRNA expression levels in lung cancer**. RNA sequencing data (ID: TCGA_LUNG_exp_HiSeqV2, *N* = 1081) from The Genome Cancer Atlas database was processed and visualized with UCSC Cancer Genomics Browser (https://genome-cancer.ucsc.edu). The color scale represents changes of gene expression: up-regulation (red), down-regulation (green) or no change (black). Data is expressed as log2 transformed RNA-seq RSEM counts normalized to log2 transformed mean of the dataset.

**Figure 4:**
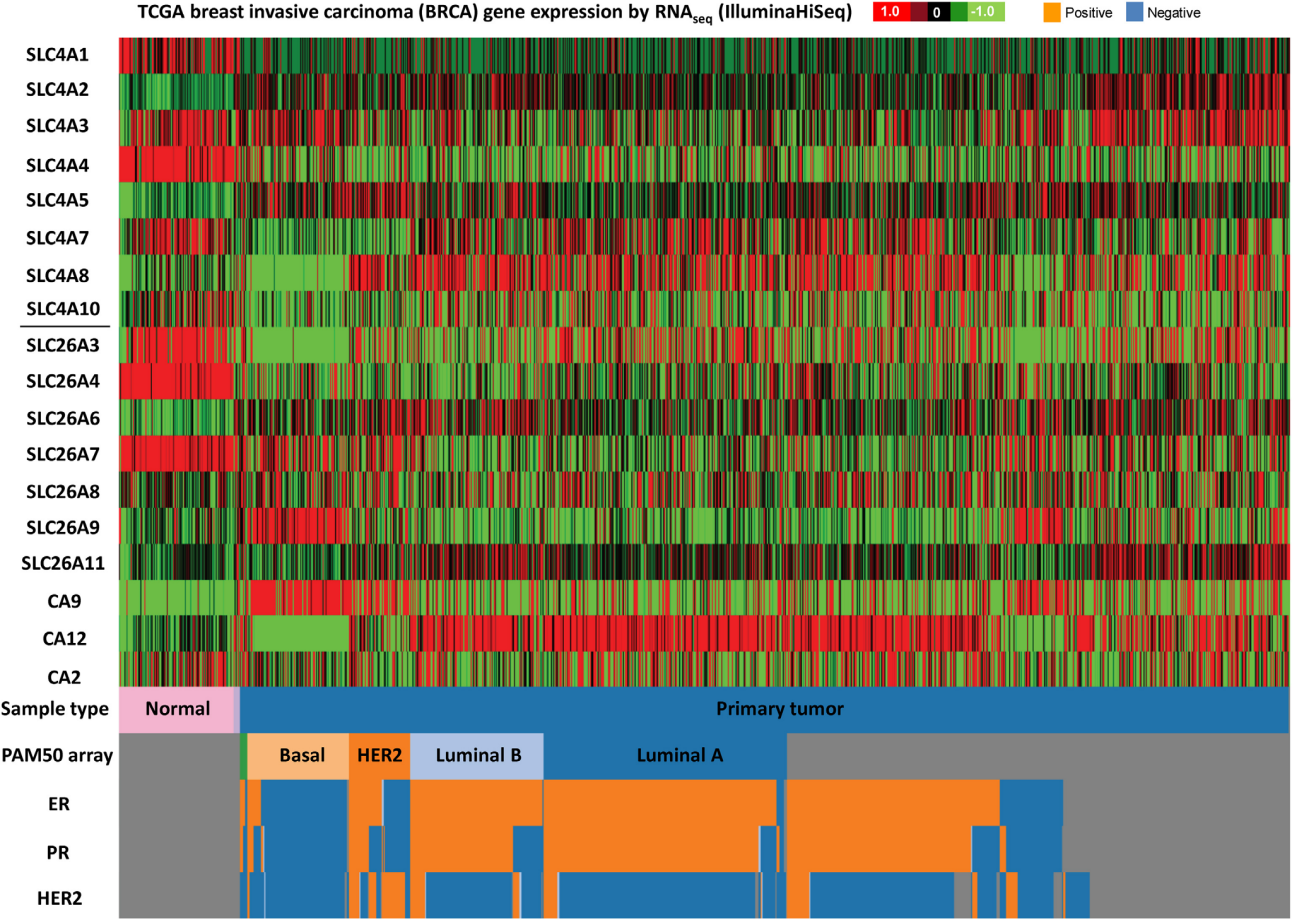
**SLC4 and SLC26 family mRNA expression levels in breast cancer**. RNA sequencing data (ID: TCGA_BRCA_exp_HiSeqV2, *N* = 1106) from The Genome Cancer Atlas database was processed and visualized with UCSC Cancer Genomics Browser (https://genome-cancer.ucsc.edu). The color scale represents changes of gene expression: up-regulation (red), down-regulation (green) or no change (black). Data is expressed as log2 transformed RNA-seq RSEM counts normalized to log2 transformed mean of the dataset.

**Figure 5:**
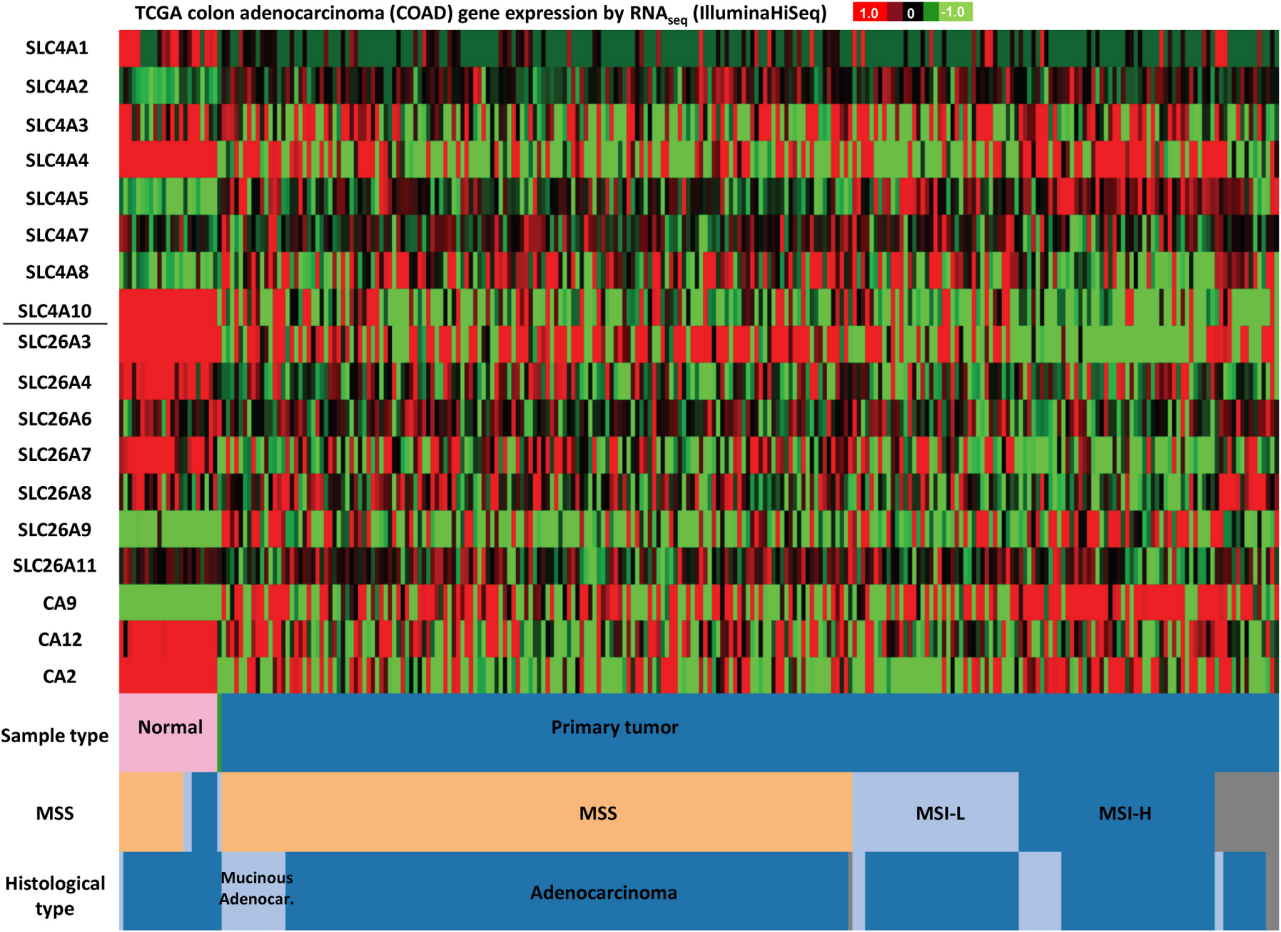
**SLC4 and SLC26 family mRNA expression levels in colorectal cancer**. RNA sequencing data (ID: TCGA_COAD_exp_HiSeqV2, *N* = 272) from The Genome Cancer Atlas database was processed and visualized with UCSC Cancer Genomics Browser (https://genome-cancer.ucsc.edu). The color scale represents changes of gene expression: up-regulation (red color), down-regulation (green) or no change (black). Data is expressed as log2 transformed RNA-seq RSEM counts normalized to log2 transformed mean of the dataset.

Figures 3–5 illustrate results from TGCA datasets on the mRNA expression profiles of SLC4 and SLC26 members (excluding those known not to carry HCO^−^_3_) and selected carbonic anhydrases (CAII, CAIX, and CAXII) in breast- (Figure 3), colon- (Figure 4), and lung cancer (Figure 5), respectively.

As seen in Figure 3, *firstly*, the mRNA expression levels of most of the SLC4 and SLC26 family members differ widely between normal and cancer tissue, with a clear trend toward upregulation of SLC4A3, SLC4A7, and SLC26A6, and downregulation of SLC4A1 in cancer compared to normal tissue. *Second*, the two major subtypes of lung cancer (squamous cell carcinoma and adenocarcinoma) differ markedly in expression of the two families of transporters. For example, SLC4A4, SLC4A8, and SLC26A9 are down-regulated in squamous cell carcinoma, but not in adenocarcinoma of the lung.

The different subtypes of breast cancer (Figure 4) show an even more heterogeneous expression pattern of SLC4 and SLC26 family transporters. Especially basal breast cancers are distinct from the other subtypes, with markedly different expression patterns of many of these proteins compared to the other subtypes, including an apparent strong and consistent upregulation of SLC26A9. However, some general trends are observed across all breast cancer subtypes: SLC4A1, SLC4A4, SLC26A3, and SLC26A4 seem to be consistently down-regulated, while SLC4A5 is up-regulated, in all breast cancer subtypes compared to normal breast tissue. Of note, the TCGA data show no consistent upregulation of SLC4A7 mRNA levels in cancer, which is at variance with the pattern indicated from protein level data in (Boedtkjer et al., 2013).

Finally, in colon adenocarcinomas (Figure 5), the TCGA data indicate that mRNA levels of SLC4A1, SLC4A4, SLC4A10, SLC26A3, SLC26A4, and SLC26A7 are down-regulated, while SLC4A2 and SLC4A5 tend to be up-regulated, compared to normal colon tissue. Thus, consistent with published findings [see section Down-Regulated in Adenoma (SLC26A3, DRA)], SLC26A3 is down-regulated in colon cancer, but furthermore, our analyses reveal that this gene is also widely down-regulated in breast cancer (Figure 4).

Interesting patterns are also seen when comparing the expression patterns of three carbonic anhydrases implicated in cancer development: CAII, CAIX, and CAXII, to each other and to that of the HCO^−^_3_ transporters. Firstly, while CAII mRNA levels are generally down-regulated through lung and colon cancer datasets (less so in breast cancer), CAIX and CAXII seem to be expressed in a highly cancer subtype-specific manner. For instance, the CAIX mRNA level is strongly increased in basal and triple-negative breast cancers, while the CAXII mRNA level is generally increased in all breast cancer subtypes except the triple-negative breast cancers. Also interestingly, the mRNA levels of both CAIX and CAXII are increased in squamous cell carcinoma of the lung, yet not in lung adenocarcinomas. Second, marked co-expression patterns are notable between specific CAs and specific HCO^−^_3_ transporters. For instance, the high levels of CAIX and CAXII in squamous cell carcinomas and low levels in adenocarcinoma is accompanied by an almost opposite expression pattern of the HCO^−^_3_ transporters, levels of which are generally high in adenocarcinoma and low in squamous cell carcinoma, the only exception being SLC4A3. Another example is the expression pattern of SLC26A9 and CAIX in breast cancer, which is strikingly similar, with high levels in basal and triple-negative breast cancers only. While this of course needs further, unequivocal validation, we hypothesize that such distinct patterns might be useful in the differentiation between specific cancer subtypes, both in breast and lung cancer.

The juxtaposition of all the HCO^−^_3_ transporter expression data in Figures 3–5 begs the question of whether there are patterns reflecting the functional categories of transporters, i.e., anion exchangers vs. Na^+^-coupled 1:1 or 1:2 HCO^−^_3_ transporters. In general, however, apart from co-expression patterns like those described above, no strong such pattern emerges. Collectively, our analyses of publicly available gene expression data confirm the notion that many HCO^−^_3_ transporters exhibit markedly altered expression patterns in cancer compared to normal tissue. However, the data does not reveal an explicit trend consistent with the notion that HCO^−^_3_ extruders would be down-regulated and HCO^−^_3_ loaders up-regulated in solid tumors. Moreover, different subtypes of the cancers studied exhibit different HCO^−^_3_ transporter expression profiles, in congruence with the known heterogeneity of genetic and epigenetic changes in cancer. Finally and importantly, these data reveal nothing about the possible posttranslational regulation of these transporters, which could result in an activity profile completely different from the expression profile. It seems likely that the specific HCO^−^_3_ transporter(s) expressed might be subject to great variance between cancer types and subtypes, whereas the functional correlate—increased net acid extrusion capacity—would be more similar. However, also at the functional level, substantial differences are expected, depending, e.g., on metabolic differences between cancer types. Future studies should focus on revealing the mechanisms of regulation of HCO^−^_3_ transporter expression and activity and the possible functional relevance of the changes observed here, for cancer development. Given the increasing evidence pointing toward a major importance of pH-regulatory ion transport dysregulation in cancer development, we hypothesize that altered HCO^−^_3_ transporter expression, if paralleled by increased activity, is likely to play a causal role in at least some cancers, and hence, that these transporters are potentially relevant as therapeutic targets.

## Conclusions and outlook

While the great majority of the research on the roles of pH_i_ dysregulation in cancer development has focused on H^+^ transport, recent evidence indicates that also HCO^−^_3_ transport is dysregulated in many cancers. The few studies available to date addressing the importance of HCO^−^_3_ transport for pH_i_ regulation and cancer cell growth show that particularly under 3D conditions, HCO^−^_3_ transport appears to play a central role. The new datamining analyses presented here suggests that many more HCO^−^_3_ transporters than those studied to date may be dysregulated in various cancers, and we hypothesize that this may have both diagnostic and therapeutic potential. However, most of the central questions remain open, including the mechanisms of transporter dysregulation, the possible causal role of the dysregulation for cancer development, and the extent to which such a role reflects the involvement of the transporters in pH_i_ regulation. Furthermore, it will be essential to validate the putative roles of HCO^−^_3_ transporters in *in vivo* models of cancer.

### Conflict of interest statement

The Associate Editor, M. Bevensee, declares that, despite having collaborated with authors S. Pederson and E. Boedtkjer, the review process was handled objectively and no conflict of interest exists. The authors declare that the research was conducted in the absence of any commercial or financial relationships that could be construed as a potential conflict of interest.
